# Cancer-Associated-Fibroblast-Derived Small Extracellular Vesicles (sEVs) in Lung Cancer Immunotherapy Resistance: Mechanistic Insights, Clinical Translations, and Current Challenges

**DOI:** 10.3390/cells15100883

**Published:** 2026-05-12

**Authors:** Shuangrui Chen, Jin Yan, Xiaochun Peng

**Affiliations:** 1 Department of Pathophysiology, School of Basic Medicine, Health Science Center, Yangtze University, Jingzhou 434020, China; 2 Laboratory of Oncology, Center for Molecular Medicine, School of Basic Medicine, Health Science Center, Yangtze University, Jingzhou 434020, China; 3 Department of Physiology, School of Basic Medicine, Health Science Center, Yangtze University, Jingzhou 434020, China

**Keywords:** cancer-associated fibroblasts, small extracellular vesicles (sEVs), lung cancer, drug-resistant, immunotherapy for lung cancer

## Abstract

**Highlights:**

**What are the main findings?**

- CAF-derived small EVs (CAF-sEVs) are strongly implicated in lung cancer immunotherapy resistance by reshaping the immune microenvironment and transferring immunomodulatory non-coding RNAs, though the extent of their independent causal contribution in vivo remains to be fully defined.
- Proteins, miRNAs, and circRNAs carried by small extracellular vesicles (sEVs) correlate with immunotherapy resistance in lung cancer patients, supporting their potential as predictive biomarkers.

**What are the implications of the main findings?**

- Targeting CAF-sEV biogenesis or their specific cargoes represents a promising but still investigational strategy to counteract immunotherapy resistance; definitive proof would require cell-type-specific genetic inhibition studies.
- sEV cargo profiling holds promise for non-invasive liquid biopsy to monitor resistance emergence, but large-scale prospective validation in longitudinal immunotherapy cohorts is required before clinical adoption.

**Abstract:**

Immunotherapy has emerged as an established clinical approach for lung cancer; however, both intrinsic and adaptive resistance mechanisms substantially constrain its therapeutic efficacy. Within the tumor microenvironment (TME), cancer-associated fibroblasts (CAFs) serve as pivotal stromal mediators of this resistance. These fibroblasts manifest their immunomodulatory effects, in part, through the secretion of small extracellular vesicles (sEVs). While multiple lines of evidence strongly implicate CAF-sEVs in immunotherapy resistance, establishing a direct causal link remains an active area of investigation. Clinically, elevated levels of specific CAF-sEV cargoes correlate with poor response in lung cancer patients. Functionally, CAF-sEVs can directly suppress T-cell activity and drive pro-resistance phenotypes via defined molecular pathways, and pharmacological inhibition of sEV secretion has been shown to attenuate resistance in preclinical models. However, the extent to which these effects are independent of other CAF-derived factors remains to be fully elucidated in vivo. This review comprehensively synthesizes the biophysical properties of CAF-derived sEVs, delineates the molecular mechanisms underpinning their role in immunotherapy resistance, critically evaluates the existing causal evidence and its limitations, and assesses their translational potential as diagnostic biomarkers and therapeutic targets—ultimately providing a conceptual framework to overcome resistance barriers in lung cancer immunotherapy.

## 1. Introduction

**Figure 1:**
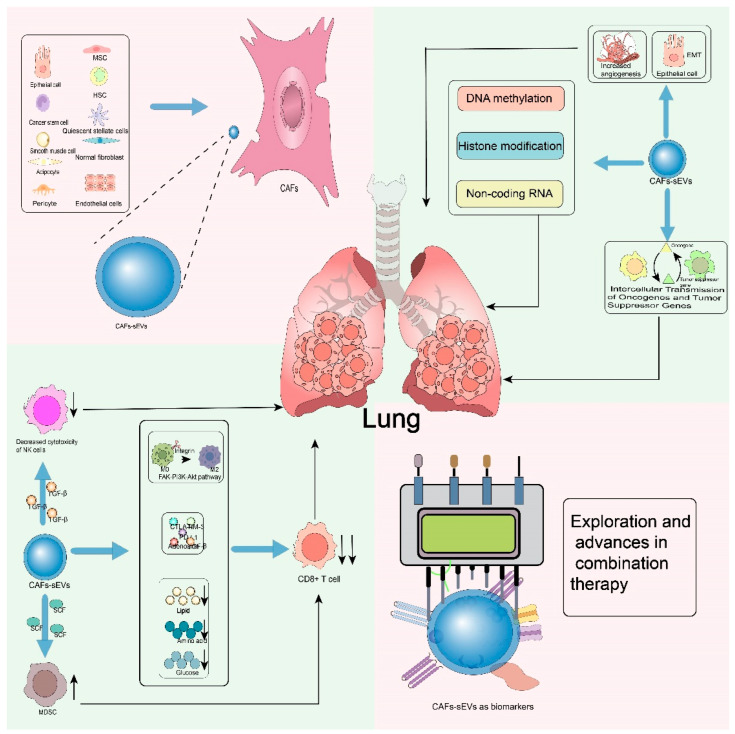
The sources, functions and potential of small extracellular vesicles (sEVs). Short arrows indicate rise and fall, long arrows represent progressive relationships, and blue arrows stand for differentiation or source.

Lung cancer is one of the most common cancers worldwide, and it is also one of the leading causes of cancer-related deaths. Immunotherapy, on the other hand, has ushered in a new era of cancer treatment and transformed the therapeutic landscape of various human malignant tumors [1,2]. In recent years, immune checkpoint inhibitors have significantly prolonged the survival of specific patient populations. However, the frequent emergence of primary and secondary resistance is a key factor limiting therapeutic efficacy [3]. Recent studies have shown that drug resistance in antitumor drugs is closely related to the tumor microenvironment (TME), especially cancer-associated fibroblasts (CAFs). Small extracellular vesicles derived from CAFs regulate the proliferation, metastasis and therapeutic resistance of non-small cell lung cancer through their carried active cargoes and can serve as non-invasive biomarkers with diagnostic potential [4,5] (Figure 1).

The key to drug resistance in tumor immunotherapy lies in the regulatory role of the tumor microenvironment (TME), a special ecosystem. As the “main battlefield” for the interaction between tumor cells and the immune system, the tumor microenvironment is a complex network composed of various cellular components (including vascular endothelial cells, immune cells, cancer-associated fibroblasts, etc.) and acellular components (such as the extracellular matrix and various cytokines), forming a dynamic balance [6]. The core mechanisms by which the tumor microenvironment mediates immune resistance include: (1) bioactive substances secreted by cancer-associated fibroblasts (CAFs) form physical and biochemical barriers; (2) the accumulation of abnormal metabolites impairs the function of effector T cells; (3) abnormal vascular networks hinder immune cell infiltration; (4) epigenetic modifications induce T-cell exhaustion. These processes collectively construct a systemic immunosuppressive microenvironment [7].

**Figure 2:**
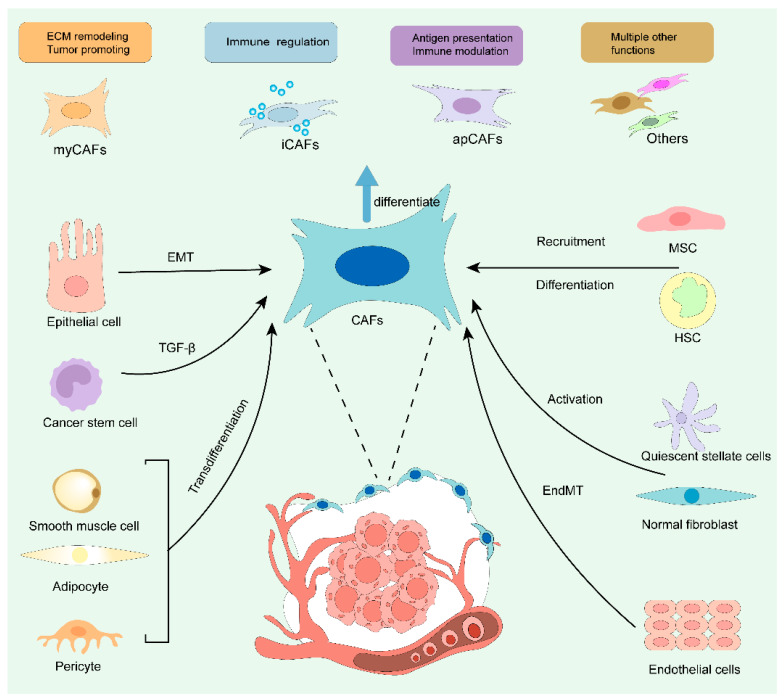
Origin and function of cancer-associated fibroblasts. Cancer-associated fibroblasts (CAFs) are primarily generated by the activation of resident fibroblasts, but can also be formed through trans-differentiation of bone marrow mesenchymal stem cells, endothelial cells, epithelial cells, and various other cell types. Cancer-associated fibroblasts can be further divided into different subtypes with distinct functions. The blue arrows represent differentiation.

Cancer-associated fibroblasts are the most active and abundant stromal components in the tumor microenvironment, and can dynamically regulate tumor progression and metastasis through interactions with cancer cells. Cancer-associated fibroblasts have diverse origins and are mainly formed by the activation of tissue-resident fibroblasts [8,9]. They also originate from the differentiation of bone marrow mesenchymal stem cells, endothelial–mesenchymal transition, or epithelial–mesenchymal transition (EMT) [10]. In addition, CAFs have other, less common origins, including pericytes, adipocytes, mesothelial cells, and smooth muscle cells [11,12,13,14,15]. Traditionally, CAFs expressing αSMA are classified as myCAFs. myCAFs are mainly involved in ECM remodeling and tumor promotion. iCAFs are characterized by elevated secretion of cytokines and chemokines, which play key roles in immune regulation. apCAFs are identified by the expression of major MHC class II molecules and CD74, and participate in antigen presentation and modulation of immune responses. CAFs, cancer-associated fibroblasts; MSCs, mesenchymal stem cells; MMT, macrophage-to-myofibroblast transition; EMT, epithelial–mesenchymal transition; EndMT, endothelial-mesenchymal transition; αSMA, α-smooth muscle actin; myCAFs, myofibroblastic CAFs; extracellular matrix (ECM); inflammatory CAFs; apCAFs, antigen-presenting CAFs; MHC, major histocompatibility complex [16] (Figure 1). The process by which fibroblasts transform into a tumor-associated phenotype is driven by environmental stress [17], such as hypoxia and reactive oxygen species, and functionally communicate between cells through secretory factors and extracellular vesicles [18]. There are two main cytokine drivers of CAF transformation: One is TGFβ, which mainly drives the transformation of fibroblasts, endothelial cells and mesenchymal stem cells into CAFs [19,20]. The other is platelet-derived growth factor-BB (PDGF-BB), which focuses on fibroblast proliferation and recruitment [21]. Elevated PDGF-BB expression has been observed in most cancers (colon cancer, brain cancer, breast cancer, prostate cancer, and lung cancer), and high stromal expression of PDGF receptor β (PDGFR-β) predicts rapid cancer progression and poor prognosis [22,23]. They collectively contribute to the formation of the reactive tumor stroma. In the TME, CAFs are an important source of exosomes [24]. They collectively contribute to the formation of the reactive tumor stroma [25] (Figure 2).

Associated fibroblasts exert pleiotropic effects through extracellular vesicle (sEVs)-mediated cell communication: they secrete chemokines (such as CXCL12 and CCL5) to recruit immune cells and myeloid-derived suppressor cells, thereby promoting tumor proliferation [26,27,28,29]. Simultaneously, they deliver angiogenic factors such as vascular endothelial growth factor (VEGF) to sustain tumor angiogenesis [30,31]. Cancer-associated fibroblast-derived extracellular vesicles can also transport cytokines (such as IL-6 and CXCL12) to activate the PI3K/Akt signaling pathway and promote bone metastasis [32]. In addition, such vesicles can downregulate *Wnt* signaling inhibitors via miR-148a [33], which activates the *Wnt* pathway to further enhance the migration ability of cancer cells. In terms of chemotherapy resistance, extracellular vesicles derived from cancer-associated fibroblasts transmit the HGF/MET signal to antagonize epidermal growth factor receptor (EGFR) inhibitors, secrete Wnt16b to inhibit apoptosis, and activate the nuclear factor-κB (NF-κB) pathway through IL-17A to maintain the drug resistance of cancer stem cells [34,35,36,37,38,39]. In summary, these mechanisms highlight the central role of cancer-associated fibroblasts and their secreted extracellular vesicles in tumor progression and therapeutic resistance. Therefore, this review elaborates on the drug resistance mechanisms mediated by extracellular vesicles and proposes novel therapeutic strategies for lung cancer (LC).

## 2. Biological Characteristics of Cancer-Associated Fibroblast Small Extracellular Vesicles (sEVs)

### 2.1. Definition and Origin of Small Extracellular Vesicles

According to IMSEV2023, small extracellular vesicles (sEVs) are a class of extracellular vesicles with a typical bilayer membrane structure, usually less than 200 nanometers in diameter, including exosomes and microvesicles [40]. Extracellular vesicles (EVs), which are intraluminal vesicles released via multivesicular bodies (MVBs), are called exosomes, with a diameter typically ranging from 30 to 150 nanometers [41]. Microparticles are extracellular vesicles (EVs) on the cell surface that are formed by direct budding from the plasma membrane. Exosomes, initially underestimated as cellular waste disposal tools, are now recognized as important participants in intercellular communication [42,43]. In the tumor microenvironment, cancer-associated fibroblasts (CAFs) are an important source of small extracellular vesicles, which can be widely distributed through easily accessible body fluids such as blood, saliva, breast milk or urine [44], which provide favorable conditions for CAFs’ involvement in tumor-related regulation [45].

### 2.2. Structure and Composition of Small Extracellular Vesicles

Small extracellular vesicles are spherical in shape and enclosed by a phospholipid bilayer, which can protect their contents (such as proteins, lipids, metabolites, messenger RNA, mitochondrial DNA, microRNAs, and other non-coding RNAs) from enzymatic degradation during transport from donor cells to recipient cells [46]. Small extracellular vesicles contain a variety of molecules, such as proteins, lipids, metabolites, messenger RNA, mitochondrial DNA, microRNA and other non-coding RNAs [45,47,48]. The complexity and heterogeneity of these components enable small extracellular vesicles to play a crucial role in intercellular communication [48]. Small extracellular vesicles contain a variety of constitutive proteins closely related to their biogenesis and structure, including members of the transmembrane protein family (CD63, CD9, CD81, and CD82), Rab guanosine triphosphatases, annexins associated with membrane fusion, chaperone proteins (heat shock protein 70 and heat shock protein 90), and tumor susceptibility gene 101 protein, which is involved in vesicle formation. In addition, small extracellular vesicles also transport cell-specific proteins that reflect their cellular origin, such as cytokines and growth factor receptors [49]. In nucleic acid components, small extracellular vesicles carry messenger RNAs screened via specific 3′ untranslated region motifs, microRNAs sorted by tetranucleotide sequences or RNA-binding proteins such as SYNCRIP, mitochondrial DNA, and a variety of non-coding RNAs [50,51,52,53]. In addition to proteins and nucleic acids, small extracellular vesicles also contain lipids such as phospholipids and cholesterol (which participate in the formation of membrane structures and lipid rafts) [54] as well as metabolites such as amino acids and nucleotides that reflect cellular metabolic status [54]. In addition, despite significant heterogeneity in the size and cargo composition, small extracellular vesicles from different sources still contain common components such as the transmembrane protein CD63.

### 2.3. Secretion and Uptake of Small Extracellular Vesicles

Small extracellular vesicles include exosomes and microvesicles that bud directly from the plasma membrane. The secretion and uptake of small extracellular vesicles are crucial for their biological functions. The secretion process occurs spontaneously, and both processes are regulated by cellular stress and activation signals [55]. Among them, exosomes are the main subject of current research and can be defined as a specific subtype of small extracellular vesicles based on their biogenetic origin (cellular origin). The biogenesis of small extracellular vesicle subsets is a tightly regulated process, which can be roughly divided into three main stages: formation of small extracellular vesicle subsets, cargo sorting, and release of small extracellular vesicle subsets [56]. First, (a) the plasma membrane invaginates to form early endocytic vesicles [51]; second, (b) early endosomes bud inward at their limiting membrane to form multivesicular bodies (MVBs) containing intraluminal vesicles (ILVs); finally, (c) multivesicular bodies fuse with the plasma membrane and release intraluminal vesicles into the extracellular space in the form of small extracellular vesicle subsets [56,57,58].

After secretion, exosomes and microvesicles are taken up by neighboring cells through endocytosis or fusion with the plasma membrane [59,60]. After internalization, exosomes can be degraded by lysosomes within recipient cells. In addition, endocytosed exosomes may fuse with endosomes and subsequently disintegrate to release their vesicular contents into the cytoplasm, or fuse with the plasma membrane to release exosomes outside the recipient cells [61,62,63].

Subpopulations of small extracellular vesicles communicate with other cells through vesicle docking and fusion, a process mediated by soluble N-ethylmaleimide-sensitive factor attachment protein receptor complexes and endosomal sorting complexes required for transport. Endosomal sorting complexes required for transport are indispensable for the biogenesis of subpopulations of small extracellular vesicles and represent a core mechanism in their biogenesis [64].

**Figure 3:**
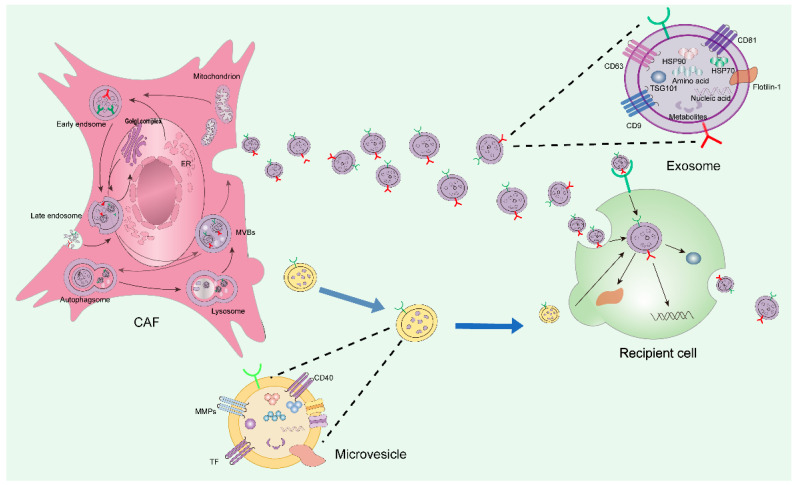
Biogenesis and structural composition of small cell vesicles. Small extracellular vesicles secreted by cancer-associated fibroblasts are composed of small microvesicles formed by direct budding and exosomes derived from the multivesicular body (MVB) endosomal pathway. After secretion, they are taken up by recipient cells through endocytosis, binding to cell surface receptors, and other means, and are degraded into various substances under the action of lysosomes. However, some small extracellular vesicles can also be excreted by recipient cells. Structurally, they all possess a bilayer membrane structure, with certain differences in specific biomarkers.

Small extracellular vesicles mainly promote signal transmission to recipient cells through three pathways: endocytosis, direct membrane fusion, and receptor–ligand interaction. Endocytosis is the main uptake pathway, which can be mediated by caveolin or lipid rafts. The internalized small extracellular vesicles can then fuse with endosomes or be degraded by lysosomes [65]. Vesicle membranes can also fuse directly with the plasma membrane, releasing their contents into the cell. Small extracellular vesicles can further bind to the plasma membrane of target cells through ligand–receptor interactions, thereby activating intracellular signaling pathways such as the protein kinase B pathway. This receptor–ligand interaction mechanism was first discovered in insect cells. Studies have shown that cultured insect cells expressing native Hedgehog and Wingless proteins can secrete small extracellular vesicles containing these two protein components. The above multiple mechanisms collectively ensure that small extracellular vesicles can accurately transmit information to target cells, thus playing an important role in intercellular communication (Figure 3).

## 3. A Framework for Evaluating Causal Evidence in Tumor Microenvironment Research

Establishing a direct causal relationship between a specific TME component and a complex phenotype such as immunotherapy resistance requires rigorous evidentiary standards that extend beyond correlation or association. Drawing upon the Bradford Hill criteria for causal inference in biological systems [66,67,68], we propose a three-tiered framework to evaluate the evidence linking CAF-derived sEVs to immunotherapy resistance:

Tier I—This encompasses two categories of observations. The first involves clinical associations between sEV cargo levels and immunotherapy resistance status in patient samples. Numerous studies have demonstrated that elevated levels of specific proteins, miRNAs, lncRNAs, and circRNAs in circulating sEVs correlate with poor response to immune checkpoint inhibitors in NSCLC patients. For instance, high plasma sEV PD-L1 levels are associated with inferior anti-PD-1 treatment outcomes, and tumor sEV miRNAs that dampen CD8^+^ T-cell function exhibit differential expression between immunotherapy responders and non-responders [69,70,71]. The second category involves in vitro phenocopying experiments: purified sEVs from resistant donor cells, when administered to sensitive recipient cells, transfer resistance-associated phenotypes, including reduced T-cell activation, enhanced macrophage M2 polarization, and activation of pro-survival signaling pathways [69,72]. While highly suggestive, Tier I evidence is inherently limited in establishing causality. First, the correlational nature of clinical studies cannot distinguish causation from epiphenomena: resistance itself may trigger compensatory changes in sEV secretion, creating a reverse causality scenario where the observed sEV alterations are a consequence rather than a cause of treatment failure [73]. Second, many in vitro phenocopying studies employ sEV concentrations that may exceed physiological levels found in the TME, and the resistance phenotype induced by exogenous sEV administration may not faithfully recapitulate the complex, multifactorial resistance that develops in vivo. Third, technical challenges in sEV isolation—particularly the co-purification of soluble proteins and lipoproteins during ultracentrifugation—can confound the attribution of observed effects specifically to sEVs rather than to co-isolated soluble mediators. These limitations underscore that Tier I evidence, while essential for hypothesis generation, requires corroboration from higher-tier experimental designs to establish causality.

Tier II—This tier encompasses studies in which purified CAF-derived sEVs—free of contaminating soluble factors—are shown to directly induce resistance-associated phenotypes in target cells (T cells, macrophages, NK cells, tumor cells). The gold-standard criteria within this tier include: (i) dose-dependent effects; (ii) ablation of the observed phenotype by sEV depletion; and (iii) recapitulation of the effect using sEVs from genetically modified CAFs. Multiple studies meeting these criteria have demonstrated that CAF-sEVs directly suppress T-cell-mediated killing via the OIP5-AS1/miR-142-5p/PD-L1 axis [74]; promote macrophage M2 polarization through GREM1-mediated FGF4/SHH signaling [75]; confer cisplatin resistance through the transfer of miR-20a [76] and miR-130a [77], which target the PTEN/PI3K-AKT pathway; and induce EMT via miR-210-mediated PTEN/PI3K/AKT activation [78]. Dose-dependent effects have been demonstrated for CAF-exosome-mediated cisplatin resistance [77], and sEV depletion via GW4869 abolishes CAF-induced EMT in recipient cells [79]. Adherence to MISEV2023 guidelines [40] is essential for ensuring the rigor of Tier II evidence.

Tier III—This represents the most stringent level of proof, requiring CAF-specific elimination of sEV biogenesis—without affecting sEV secretion by other cell types—to prevent or reverse immunotherapy resistance in an intact tumor microenvironment. The definitive experiment would employ conditional knockout of Rab27a or nSMase2 in αSMA-expressing CAFs within immunocompetent lung cancer models. Currently, no published study has achieved this, representing the single most substantive knowledge gap in the field. However, principled support for feasibility exists: Gargiulo et al. [80] demonstrated that Rab27a/b knockout abrogated sEV secretion and restored CD8^+^ T-cell function in a leukemia model, with rescue by exogenous sEV administration; mammary carcinoma micrometastases have been shown to switch from Rab27-dependent to nSMase2-dependent sEV production to generate invasive microenvironments [81]; and NOX4 inhibition “normalized” CAFs and restored immunotherapy response in CAF-rich murine tumors (TC1, MC38, and 4T1) [82]. The field requires CAF-specific Rab27a or Smpd3 mice crossed with FAP-Cre or αSMA-CreER^T2 drivers—plus sEV rescue controls—to definitively test whether the selective elimination of CAF-sEVs restores immunotherapy sensitivity.

Throughout this review, we annotate the evidence discussed according to this framework, explicitly identifying which tier each study addresses and what residual uncertainties remain. This approach reveals a critical pattern: while Tier I and Tier II evidence is abundant and compelling, Tier III evidence remains sparse, representing a pivotal knowledge gap that must be addressed before CAF-sEVs can be confidently designated as direct causal mediators of immunotherapy resistance in vivo.

## 4. The Basic Mechanism of CAF-sEV-Mediated Immune Resistance in Lung Cancer CAFs

Unlike traditional therapies such as surgery, chemotherapy and radiotherapy, immunotherapy targets tumors directly. Cancer immunotherapy harnesses the immune system to fight against tumors. Generally speaking, immunotherapy is mainly divided into four major categories [16]: (a) Immune checkpoint inhibitors (ICIs) are the current mainstream approach. The discovery of ICIs has made checkpoint inhibitors promising for curing cancer, such as the anti-PD-L1 drugs nivolumab and pembrolizumab [83]. (b) Chimeric Antigen Receptor (CAR) T-cell therapy. CAR-T cells are autologous genetically engineered T cells that directly mediate cancer recognition and subsequent killing by binding to cancer antigen targets through the CAR, representing an innovative synthetic immunity that bypasses the classic cancer-immune cycle [84]. (c) Cytokines, such as interferon-alpha (IFN-α) and interleukin-2 (IL-2), as the first clinically used immunomodulators for cancer therapy, regulate the tumor microenvironment by enhancing the immune response [85]. (d) Therapeutic vaccines enhance tumor-specific T cells by delivering cancer antigens and adjuvants. They are effective as monotherapy when immunosuppression is not significant [86,87]. Extracellular vesicles can facilitate crosstalk between tumors and stromal cells. Exosomes have emerged as an important communication mechanism among different cell types in the tumor microenvironment (TME) [88]. CAF-derived EVs play a crucial role in mediating cancer chemoresistance through complex mechanisms and signaling pathways [89,90,91]. CAF-derived EVs have been extensively implicated in cancer chemoresistance through a variety of mechanisms and signaling pathways. However, as elaborated in Section 2, the majority of current evidence falls within Tiers I–II of our causal framework. Below, we systematically review the mechanistic pathways through which CAF-sEVs are proposed to contribute to immunotherapy resistance, while explicitly noting the evidence tier and the limitations inherent in each line of investigation.

### 4.1. Immune Checkpoint Modulation: CAF-sEVs as Carriers of Immunosuppressive Molecules Impairing T-Cell Function

CAF-sEVs carry molecules such as PD-L1, TGF-β and adenosine, directly killing or paralyzing CD8^+^ T cells [92]. CAF-sEVs can serve as natural carriers of immunosuppressive molecules, delivering key molecules such as PD-L1, TGF-β, and adenosine to the tumor microenvironment, directly leading to functional exhaustion, metabolic paralysis, and even apoptosis of CD8^+^ T cells. Among them, PD-L1 binds to PD-1 on the surface of T cells, blocking the TCR signaling pathway [93]. Extracellular vesicles secreted by cancer-associated fibroblasts carry interleukin-8 (IL-8), which can directly chemotactically regulate the infiltration of regulatory T cells into the tumor microenvironment. Regulatory T cells mediate drug resistance by suppressing anti-tumor immune responses [94].

TGF-β activates the Smad pathway to induce T-cell anergy; adenosine inhibits glycolysis via the A2A receptor, forming a multiple immunosuppressive network and mediating resistance to immunotherapy [95].

Prostaglandin E2 (PGE2) in vesicles can induce the differentiation of CD4^+^ T cells into regulatory T cells, enhancing immunosuppressive effects [96]. In addition to PD-L1, small extracellular vesicles derived from cancer-associated fibroblasts also carry other key co-inhibitory molecules. Clinical studies have shown that the levels of TIM-3 and Galectin-9 in small extracellular vesicles in the plasma of lung cancer patients are significantly elevated, and their expression levels are closely associated with tumor volume, metastatic burden, and poor prognosis. Mechanistic studies have revealed that the binding of TIM-3 to Galectin-9 can interfere with calcium signaling in natural killer cells (NK cells) and CD8^+^ T cells, and synergize with PD-L1 to suppress the activation of immune cells [97], further inhibiting the initial activation of T cells. In addition, cytotoxic T-lymphocyte-associated antigen 4 (CTLA-4) in small extracellular vesicles derived from cancer-associated fibroblasts can competitively bind to B7 molecules on the surface of antigen-presenting cells (APCs), block the CD28-B7 co-stimulatory signal, and thereby suppress the initial activation of T cells. These findings reveal the multiple regulatory roles of small extracellular vesicles derived from cancer-associated fibroblasts in tumor immune escape, providing more potential strategies for tumor therapy. On the one hand, extracellular vesicles secreted by cancer-associated fibroblasts carry interleukin-8 (IL-8), which can directly chemoattract and regulate the infiltration of regulatory T cells into the tumor microenvironment; regulatory T cells mediate drug resistance by inhibiting anti-tumor immune responses [54]. Meanwhile, prostaglandin E2 (PGE2) in the vesicles can induce the differentiation of CD4^+^ T cells into regulatory T cells, enhancing the immunosuppressive effect [55]. On the other hand, stem cell factor (SCF) in extracellular vesicles derived from cancer-associated fibroblasts can activate the c-Kit signaling pathway and promote the proliferation of myeloid-derived suppressor cells. Myeloid-derived suppressor cells further secrete interleukin-10 (IL-10) and transforming growth factor-β (TGF-β), inhibiting the function of CD8^+^ T cells [98]. In addition, prostaglandin E2 in vesicles can enhance the immunosuppressive activity of myeloid-derived suppressor cells through the STAT3 pathway. Furthermore, regulatory T cells and myeloid-derived suppressor cells form a positive feedback loop in the tumor microenvironment, and transforming growth factor-β secreted by regulatory T cells can promote the differentiation of myeloid-derived suppressor cells [96]. Myeloid-derived suppressor cells induce the expansion of regulatory T cells through arginase-1 (Arg-1). The two work together to establish an immunosuppressive microenvironment, ultimately leading to the failure of chemotherapeutic drugs such as oxaliplatin.

As the invasive characteristics of malignant tumors continue to intensify, their tendency to develop distant metastasis also increases accordingly [99]. This process requires the establishment of a metastatic microenvironment at distant sites, and tumor epithelial cells must undergo epithelial mesenchymal transition (EMT) [76].

**Figure 4:**
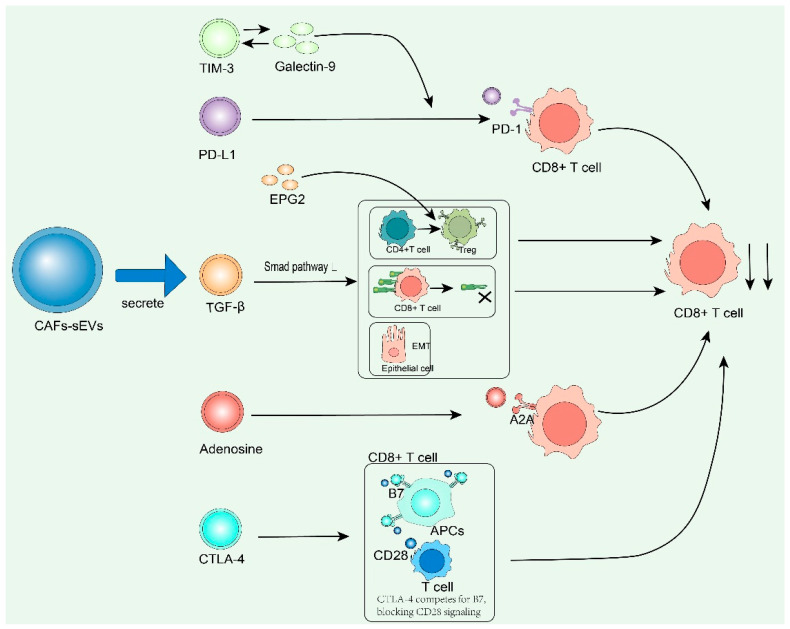
Small extracellular vesicles secreted by cancer-associated fibroblasts carry immune checkpoint molecules and can directly inhibit T-cell function.

In the transforming growth factor-β/Smad signaling pathway, small extracellular vesicles (sEVs) mediate the delivery of transforming growth factor-β, activate Smad2/3 to induce epithelial–mesenchymal transition, and downregulate major histocompatibility complex class I molecules, thereby reducing the recognition ability of T cells (Figure 4).

### 4.2. Macrophage Polarization and NK Cell Modulation: Proposed Roles of CAF-sEVs

CAF-sEVs can drive the polarization of tumor-associated macrophages towards the immunosuppressive M2 phenotype through an indirect mechanism that does not directly regulate the endogenous pathways of macrophages; polarized macrophages highly express PD-L1 and secrete large amounts of VEGF, further promoting angiogenesis, inhibiting T-cell function, and ultimately mediating tumor immune escape. CAF-sEVs can regulate macrophage polarization towards the M2 type through multiple indirect pathways. On the one hand, by depositing matrix proteins such as collagen, fibronectin, and POSTN and remodeling the stiffness and structure of the ECM, macrophages sense physical signals via integrins and polarize towards the M2 phenotype [100,101], in which POSTN can bind to integrins on the macrophage surface (without internalization) to activate BMP4 signaling and trigger the FAK-PI3K-Akt pathway, thereby promoting M2 polarization [102]. On the other hand, as an intermediary, it first acts on tumor cells or other immune cells, inducing them to secrete M2-type polarization factors or inhibiting the secretion of IFN-γ by T cells and dendritic cells to relieve the suppression of M2 polarization [103]. For example, lung cancer cells can first be activated to secrete FGF4, and then indirectly induce M2 polarization of macrophages through the FGF4/SHH pathway [25]. Meanwhile, sEVs can also transport metabolites such as lactic acid, adenosine, and ketone bodies [104]. It causes acidification, hypoxia and glucose depletion in TME, thereby activating the HIF-1α pathway in macrophages and upregulating M2 markers such as CD206 and Arg-1 [105]. In addition, CAF-derived sEVs can also carry chemokines such as CCL2 and CXCL12 to recruit circulating monocytes into the TME, and induce their directional differentiation into M2-type macrophages through M-CSF.

**Figure 5:**
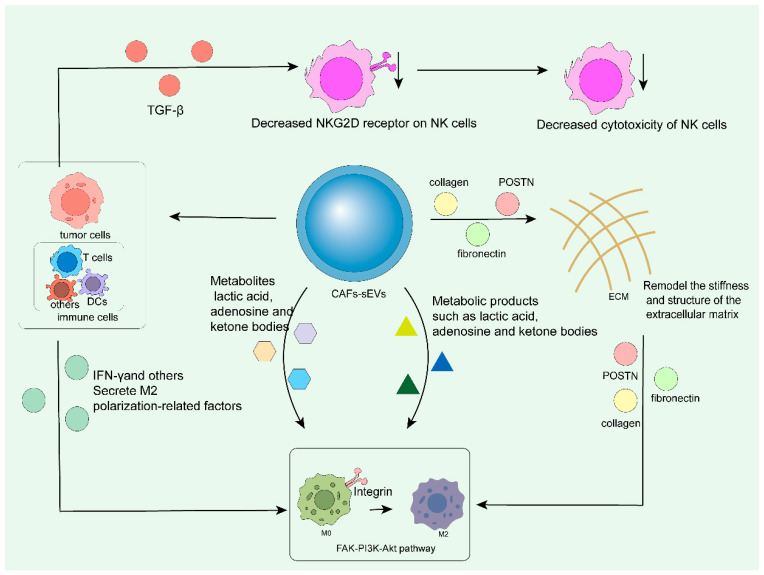
Small extracellular vesicles secreted by cancer-associated fibroblasts: 1. Act on tumor cells and immune cells (e.g., inhibiting the secretion of interferon-γ by T cells and CD cells and relieving the inhibition), inducing these cells to secrete M2 polarization factors to promote M2 polarization; 2. transport metabolic substances, activate hypoxia, and facilitate polarization; 3. secrete chemokines to recruit monocytes and induce their differentiation into M2-type macrophages; 4. remodel the stiffness and structure of the extracellular matrix (ECM) via matrix proteins, where integrins on the surface of M0 macrophages sense matrix proteins and activate the FAK-PI3K-Akt pathway, driving the polarization of M0 macrophages toward the M2 phenotype. In addition, transforming growth factor-β (TGF-β) secreted by tumor cells indirectly suppresses the cytotoxicity of natural killer (NK) cells.

CAF-sEVs significantly inhibit the anti-tumor activity of NK cells through a variety of mechanisms, remodel the tumor immune microenvironment, and then lead to the enhancement of tumor drug resistance. These mechanisms mainly include PD-L1-pathway-mediated immunosuppression and the regulation of cytokines and signaling pathways. MiR-92 carried by CAF-sEVs can promote the nuclear translocation of Yap1, thereby upregulating PD-L1 expression on the surface of tumor cells [106,107,108]. Tumor-derived TGF- β can downregulate the expression of NKG2D receptor on the surface of NK cells, thereby inhibiting their cytotoxicity [109,110]. The interaction between CAFs and tumor cells may also enhance the secretion of TGF-β and other factors, further indirectly inhibiting the activity of NK cells [111,112]. HLA-G or TGF-β carried by sEVs can also downregulate NK cell-surface-activating receptors (such as NKG2D) and weaken their killing ability [113,114]. Tumor cell-derived exosomes significantly reduce NK cell cytotoxicity by downregulating NKG2D on the surface of natural killer (NK) cells, thereby promoting immune escape and malignant progression of tumor cells [102] (Figure 5).

### 4.3. Metabolic Reprogramming: CAF-sEVs Contribute to Metabolic Remodeling of the TME

**Figure 6:**
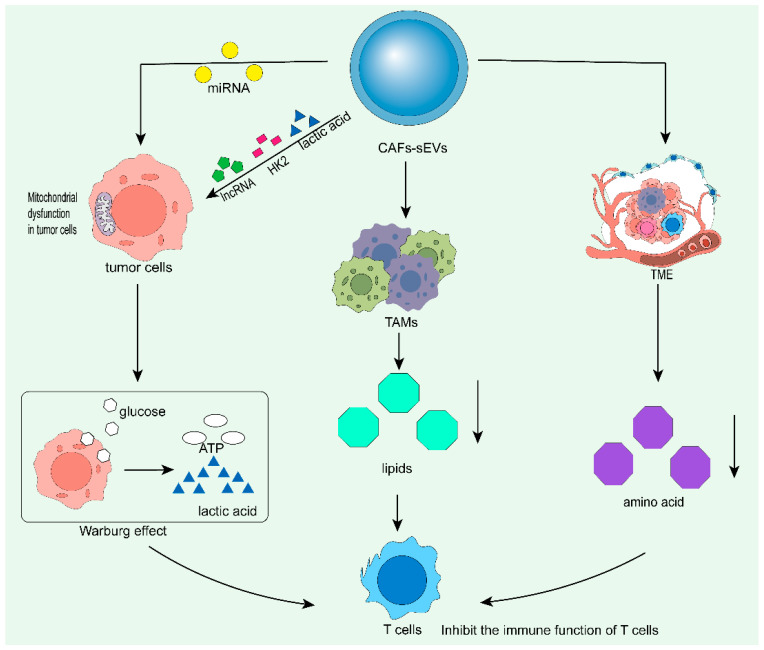
Cancer-associated fibroblasts ultimately inhibit T cells through the metabolic programming of proteins, lipids, and amino acids.

Cancer-associated fibroblast (CAF)-derived small extracellular vesicles (sEVs) can drive metabolic reprogramming and establish an immunosuppressive tumor microenvironment by regulating metabolic pathways of tumor cells and immune cells. Tumor-associated macrophages (TAMs) undergo significant metabolic reprogramming, characterized by activated glycolysis and remodeled tricarboxylic acid (TCA) cycle. They utilize alternative metabolites such as glutamine to sustain the TCA cycle, abnormal fatty acid (FA) synthesis and nitrogen metabolism, which further promotes functional reprogramming of TAMs and alters the secretion profile of cytokines and angiogenic factors. Ultimately, these changes facilitate tumor growth, suppress immune effector functions, and accelerate tumor progression and metastasis [104]. In terms of glucose metabolism, TAMs rely heavily on glycolysis for energy supply, and this characteristic is directly involved in their immunosuppressive functional reprogramming miRNAs such as miR-22 and let-7a carried by CAF-sEVs can target and inhibit key genes of mitochondrial oxidative phosphorylation (OXPHOS) (ATP synthase, COXI, and CYTB), causing mitochondrial dysfunction in tumor cells, thereby triggering the Warburg effect, manifested as an increase in extracellular acidification rate (ECAR), enhanced glucose uptake and massive lactate secretion [115]. Meanwhile, CAF-sEVs can directly transport lactate, hexokinase 2 (HK2) and long non-coding RNA SNHG3, further enhancing the glycolytic activity of tumor cells and inducing acidosis in the microenvironment, thereby directly inhibiting the mitochondrial function of infiltrating T cells [116]. In terms of lipid metabolism, TAMs exhibit enhanced fatty acid synthesis, uptake and storage capabilities. Among these, upregulated arachidonic acid metabolism, Cox2 expression and increased prostaglandin E2 (PGE2) secretion are typical metabolic characteristics of tumor-infiltrating macrophages [104]. In terms of amino acid metabolism, M2-type tumor-promoting TAMs exhibit a significant increase in glutamine consumption, accompanied by elevated levels of uridine diphosphate N-acetylglucosamine intermediates. L-arginine metabolism is also involved in the functional regulation of TAMs, with M1-type macrophages mainly relying on the nitric oxide synthesis pathway, while M2-type macrophages prefer the arginase metabolic pathway; in addition, abnormal metabolism of L-arginine, cysteine and tryptophan is an important mechanism underlying the immunosuppressive function of myeloid-derived suppressor cells (MDSCs) [103]. The aforementioned metabolic abnormalities consume large quantities of essential amino acids in the tumor microenvironment, resulting in nutrient deprivation of T cells and ultimately weakening their anti-tumor immune response capabilities [88]. Exosomes derived from CAFs inhibit mitochondrial oxidative phosphorylation, which is associated with a compensatory increase in glycolysis (i.e., the Warburg effect [88,117]. In particular, exosomes can reduce the percentage of glucose converted to α-ketoglutarate and redirect it toward lactate instead. Mitochondrial respiratory capacity is inhibited by exosomes secreted by CAFs [88] (Figure 6).

### 4.4. Cross-Activation of Drug Resistance-Related Pathways

In the tumor microenvironment, sEVs orchestrate malignant progression through a bidirectional signaling network. On one hand, they augment PD-L1 expression via the IGF-1-PI3K/AKT/mTOR cascade, thereby fostering immune evasion and chemoresistance. On the other hand, under hypoxic stress, they instigate VEGF-mediated angiogenesis through HIF-1α and potentiate Notch signaling to activate epithelial–mesenchymal transition (EMT). Consequently, this interplay collectively accelerates tumor progression and metastasis.

### 4.5. Transmission of Epigenetic Modifications

Epigenetic DNA modifications of oncogenes or anti-cancer genes are crucial for the occurrence, proliferation, and metastasis of many tumors. Dynamic variations in DNA methylation are one of the most common factors affecting the transcription of oncogenes and anti-cancer genes [118]. Epigenetic changes, especially DNA methylation patterns, have emerged as promising biomarkers for various cancers, including lung cancer [119]. An important mechanism by which sEVs epigenetically regulate drug resistance is through the transfer of methyltransferases [120]. Evidence that sEVs carry DNA methyltransferases and their impact on the drug resistance of recipient cells indicates that sEVs can alter the methylation status of recipient cells by transferring DNA methyltransferase mRNA or related proteins, thereby inducing or enhancing drug resistance.

Specifically, in temozolomide-resistant cells in glioma models, the mRNA level of MGMT (O-6-methylguanine DNA methyltransferase) is increased, and the level of MGMT mRNA in their sEVs is also synchronously increased, while the level of MGMT mRNA in sensitive cells and their sEVs is relatively low [121]. Furthermore, in ovarian cancer research, the level of DNA methyltransferase 1 (DNMT1) transcripts in cisplatin-resistant cells is positively correlated with the level of this transcript in sEVs [122]. These studies indicate that sEVs can not only carry methyltransferase-related molecules, but also that this transfer can induce recipient cells to acquire drug resistance.

In the tumor microenvironment, interactions between cancer cells can also affect drug resistance. Glioma cells can stimulate astrocytes to transform into “reactive astrocytes,” and the level of MGMT in sEVs released by these reactive astrocytes is significantly increased. After sensitive MGMT-negative glioma cells take up these sEVs, their resistance to TMZ is enhanced [123]. This directly proves that sEV-mediated transfer of methyltransferases can induce drug resistance in recipient cells.

In recipient cells, sEV-mediated transfer of DNA methyltransferases can induce cellular drug resistance. For example, in ovarian cancer research, after cisplatin-sensitive cells receive sEVs from drug-resistant cells through co-culture, their resistance to cisplatin is significantly enhanced. In vivo experiments also show that injection of sEVs carrying DNMT1 can promote tumor growth, while the sEVs inhibitor GW4869 can reverse this drug resistance phenomenon, excluding interference from other factors [124]. In the study of gliomas, the transfer of sEV-associated MGMT directly causes sensitive cells to acquire a drug-resistant phenotype, which is closely related to “cross-resistance” in the tumor microenvironment.

In addition, sEVs can also carry other cargoes to regulate methyltransferases in recipient cells, thereby indirectly affecting drug resistance. For example, sEVs released by CAFs carry miR-29b. After being transferred to hepatocellular carcinoma (HCC) cells, they can inhibit DNA methyltransferase 3b (DNMT3b) in HCC cells, thereby altering their phenotype. SEVs carry DNMT1 to induce methylation silencing of tumor suppressor genes (such as CDKN2A) and simultaneously reduce the methylation of the PD-L1 promoter.

Histone modifications and the accompanying methylation are associated with the pathogenic expression of tumor-related genes; therefore, chromatin remodeling is another potentially detectable cancer biomarker [125]. SEVs deliver EZH2, which inhibits antigen-presenting genes (such as HLA-DRA) through H3K27me3. In the cancer microenvironment, sEVs may be involved in the occurrence, proliferation, and metastasis of cancer. It has been reported that a cancer cell line, namely the G26/24 oligodendroglioma cell line, can release extracellular vesicles containing differentiation-specific linker histone H1°, while normal astrocytes do not. It is a multifaceted process driven by intrinsic tumor mechanisms, microenvironmental crosstalk, and intercellular communication, among which sEVs have become key mediators. These extracellular vesicles not only promote tumor progression and metastasis but also coordinate resistance to chemotherapy, targeted therapy, and immunotherapy by transferring substances such as miRNAs and proteins between cancer and stromal cells.

### 4.6. Intercellular Transmission of Oncogenes and Tumor Suppressor Genes

Furthermore, sEVs can also act as “genetic messengers” to enable the intercellular transfer of oncogenes and tumor suppressor genes, thereby reshaping the tumor immune microenvironment. On one hand, mutant oncogenes can be transferred to recipient cells via sEVs: tumor-derived sEVs carry mutant KRAS or EGFRvIII, and after entering target cells, they continuously activate the MAPK signaling cascade, which not only drives malignant cell proliferation but also transcriptionally upregulates PD-L1, enhancing immune evasion. On the other hand, tumor suppressor genes are silenced due to sEV-mediated epigenetic interventions: sEVs enriched with miR-148b are delivered to recipient cells, where they target and degrade the DNA methyltransferase DNMT1, leading to increased methylation levels in the PTEN promoter region, subsequent silencing of PTEN expression, and dysregulation of the PI3K/AKT pathway, which further accelerates tumor progression and impairs immune surveillance. By simultaneously “delivering oncogenes” and “erasing tumor suppressor genes,” sEVs establish a dual pro-cancer channel between cells, laying the molecular foundation for tumor drug resistance and metastasis.

## 5. Therapeutic Potential of Small Extracellular Vesicles

Histologically, lung cancer can be classified into small cell lung cancer (SCLC) and non-small cell lung cancer (NSCLC), among which non-small cell lung cancer accounts for more than 80%, mainly including subtypes such as adenocarcinoma of the lung (AC) and squamous cell carcinoma (SCC). Lung cancer immunotherapy, represented by PD-1/PD-L1 inhibitors, CAR-T cell therapy, and CAR-M cell therapy, has become a key strategy to reverse tumor immunosuppression and improve patient prognosis. However, issues such as insufficient tumor infiltration, therapeutic resistance, and disordered tumor immune microenvironment still significantly limit the clinical efficacy of immunotherapy. Small extracellular vesicles (sEVs) derived from the pathological microenvironment of lung cancer are expected to serve as diagnostic markers and therapeutic-efficacy monitoring indicators for lung cancer immunotherapy.

### 5.1. sEVs Can Serve as Diagnostic Biomarkers

Cancer is a highly heterogeneous disease involving multiple components, so the development of effective cancer biomarkers is a crucial method for indicating cancer status and monitoring cancer progression [126]. Liquid biopsy is a novel approach that collects biological fluid samples, offering more opportunities for early cancer diagnosis, prognosis prediction, and therapeutic efficacy evaluation [126].

Given that exosomes are secreted by living cells, specific contents within exosomes may reflect the pathophysiological state of their parent cells, making them useful biomarkers for the dynamic monitoring of disease progression [127].

#### 5.1.1. Protein

Lung cancer-derived small extracellular vesicles (sEVs) carry a diverse set of proteins that hold promise as diagnostic, prognostic, and predictive biomarkers for non-small cell lung cancer (NSCLC). Notably, sEV-associated PLA2G10 is significantly upregulated in NSCLC patients compared with healthy individuals, and its expression correlates with shorter overall and recurrence-free survival, making it a robust prognostic biomarker [128]. Meanwhile, sEV-expressed GCC2 varies considerably across pathological stages, exhibiting high sensitivity and specificity for early-stage NSCLC detection and supporting its utility in screening asymptomatic populations [128]. The GCC2-ALK fusion protein identified in NSCLC drives constitutive activation of the ALK pathway, which can be effectively inhibited by ALK inhibitors such as crizotinib and ceritinib, highlighting GCC2’s dual potential as a diagnostic marker and therapeutic target [100]. Proteomic profiling has further revealed distinct levels of sEV-associated and circulating lipopolysaccharide-binding protein (LBP) between metastatic and non-metastatic NSCLC patients, suggesting LBP as a potential biomarker for metastasis risk stratification [129]. In terms of therapeutic resistance, hypoxia-induced sEVs deliver PKM2 to cisplatin-sensitive NSCLC cells, conferring chemoresistance and establishing sEV-PKM2, both as a key mediator of resistance and a predictive biomarker for cisplatin response. Epidermal growth factor receptor (EGFR), a receptor tyrosine kinase involved in cell growth and survival, is frequently overexpressed or mutated in NSCLC and associated with resistance to EGFR tyrosine kinase inhibitors (EGFR-TKIs) [130]. Consistently, sEVs derived from EGFR-TKI-resistant cancer cells transfer EGFR and its mutants to recipient cells, activating downstream signaling and bypassing drug inhibition; these sEV-associated EGFR variants may thus serve as biomarkers of TKI resistance [131]. Exosomes enriched in lncRNA FOXD3-AS1 also promote 5-fluorouracil (5-FU) resistance, proliferation, and invasion in lung cancer cells by interacting with ELAVL1 to activate the PI3K/Akt signaling pathway [132]. Fibrinogen-5 activates the Src-STAT3 pathway in CAFs and cancer cells by binding to the integrin αVβ5 receptor, thereby downregulating ACSL4 to impair irradiation-induced ferroptosis. Moreover, fibrinogen-5 in CAFs can serve as a predictive biomarker for radiotherapy efficacy in NSCLC [133]. In addition, FAM83F derived from CAF exosomes interacts with KIF23 in non-small cell lung cancer cells. It upregulates KIF23 expression by activating the *Wnt*/β-catenin signaling pathway, thereby driving the proliferation, migration, invasion and radioresistance of NSCLC cells. Moreover, KIF23 is highly expressed in NSCLC tissues and is correlated with poor patient prognosis [134].

#### 5.1.2. RNA

miRNAs can regulate immune checkpoints in lung cancer, influence tumor progression, and serve as biomarkers in lung cancer immunotherapy research. Moreover, the 76-gene epithelial–mesenchymal transition (EMT) signature, based on mRNA expression in non-small cell lung cancer (NSCLC), predicts responses to targeted and chemotherapy drugs in both cell lines and patient tumors. Numerous studies have found that many primary human NSCLC tumors express PD-L1, with its expression correlating with the tumor mesenchymal phenotype regulated by miR-200 levels [135]. A serum-based four-miRNA signature comprising miR-193b, miR-301, miR-141, and miR-200b can distinguish between healthy individuals and NSCLC patients [136]. Small extracellular vesicles’ miRNAs are closely associated with NSCLC occurrence, progression, and metastasis. They not only promote angiogenesis but also function as biomarkers for lung cancer diagnosis, prediction, and prognosis. Hu et al. summarized the role of small-extracellular-vesicle miRNAs as biomarkers in lung cancer. Specifically, small-extracellular-vesicle miR-451a, miR-21, and miR-4257 exhibit aberrant overexpression in NSCLC patients and correlate with tumor progression, recurrence, and poor prognosis [57]. Analysis of plasma-derived exosomes from 45 NSCLC patients and 31 controls revealed that small-extracellular-vesicle miR-126 is upregulated and serves as a diagnostic biomarker for NSCLC [137]. Elevated circulating small-extracellular-vesicle-miR-23a levels in lung cancer patients’ serum positively correlated with pro-angiogenic activity, suggesting its potential as a diagnostic biomarker for lung cancer [57]. In cisplatin-resistant NSCLC tissues and cells, the expression of miR-369-3p is upregulated and correlated with malignant phenotypes. Mechanistic studies have revealed that CAF-derived EVs carry miR-369, which activates the MAPK signaling pathway by directly binding to NF1. Functional experiments have verified that inhibiting miR-369 can markedly weaken the proliferation, migration and invasion abilities of LUSC cells in vitro and reduce the incidence of pulmonary and hepatic metastasis in vivo [138].

Long non-coding RNAs (LncRNAs) are defined as non-coding RNAs exceeding 200 nucleotides, participating in cellular processes including cell cycle regulation, apoptosis, and genomic stability [139]. In lung cancer, LncRNAs primarily influence tumor progression by modulating immune system components and T-cell activity within the tumor microenvironment (TME). Specifically, T cells are suppressed by myeloid-derived suppressor cells (MDSCs), while LncRNAs regulate MDSC-mediated immunosuppression in the TME. Reduced HOTAIRM1 expression in lung cancer patients’ peripheral blood cells was observed; its overexpression diminished MDSC immunosuppressive properties, enhancing anti-tumor immunity [136]. Additionally, upregulated LINC00301 in non-small cell lung cancer (NSCLC) reduces CD8^+^ T-cell levels via TGF-β targeting, accelerating NSCLC progression. Exosomal LncRNAs—novel regulatory molecules—are selectively packaged into sEVs, acting as intercellular messengers that govern tumor growth, metastasis, angiogenesis, and TME remodeling [140,141]. These exosomal RNAs exhibit direct relevance to NSCLC pathogenesis. For instance, Zang et al. reported elevated UFC1 expression in NSCLC tumor tissues, serum, and serum-derived sEVs, correlating with tumor invasion [139,142]. Critically, sEV-delivered UFC1 bound EZH2, downregulated PTEN, and activated the PI3K/Akt pathway to promote NSCLC development [143]. In mouse xenograft models, MALAT1 ASO served as a potential therapeutic approach targeting cancer-related lncRNAs and can effectively inhibit lung cancer metastasis [144]. Similarly, the cancer-associated LncRNA-ATB demonstrated abnormal expression in lung, hepatocellular (HCC), colorectal (CRC), and gastric cancers. It drove tumor progression by competitively binding the miR-200 family to induce epithelial–mesenchymal transition (EMT) [145]. sEV lncRNA OIP5-AS1, derived from CAF, promotes the progression of lung cancer by regulating the miR-142-5p/PD-L1 pathway [76].

Circular RNAs (circRNAs) critically regulate the development, progression, and immunotherapy response of non-small cell lung cancer (NSCLC). CircNDUFB2 is significantly downregulated in NSCLC tissues and negatively correlated with malignant characteristics, including enlarged tumor volume, lymph node metastasis, and advanced clinical stage. Furthermore, it stimulated anti-tumor immunity throughout NSCLC development [146]. Distinct circRNAs exert subtype-specific regulatory effects: in lung adenocarcinoma (LUAD), circPRKCI upregulates E2F7 expression by competitively binding with miR-545 and miR-589, thereby promoting tumorigenesis in lung squamous cell carcinoma (LUSC), a TP63-associated circRNA upregulates FOXM1 by competitively sponging miR-873-3p, driving tumor cell proliferation through immune response modulation. Small extracellular vesicle (sEV)-mediated circRNA delivery contributes to NSCLC pathogenesis and immune regulation. Wang et al. identified upregulation of hsa-circRNA-002178 in LUAD tissues; sEV-mediated delivery to CD8^+^ T-cells induces PD-1 expression, resulting in T-cell exhaustion [147]. Additionally, tumor-derived sEV-carried circUSP7 inhibits CD8^+^ T-cell secretion of IFN-γ, TNF-α, granzyme B, and perforin, impairing anti-tumor function and potentially compromising anti-PD-1 therapy efficacy [148]. circ_PIP5K1A and ABCC1 were highly expressed, and miR-101 was lowly expressed in NSCLC. Knockdown of small-extracellular-vesicle circ_PIP5K1A suppressed cell proliferation, migration and invasion and elevated apoptosis and cisplatin sensitivity via regulating miR-101 and ABCC1, suggesting that small-extracellular-vesicle circ_PIP5K1A might be a promising application in NSCLC diagnosis and treatment [149] (Table 1).

### 5.2. sEVs Serve as Nanoscale Drug Delivery Vehicles for Targeted Therapy

In lung cancer immunotherapy, therapeutic molecules such as immunomodulators, CAR mRNA, and immune checkpoint inhibitors can be loaded into sEVs, and precise targeting of lung cancer tumor tissues and related immune cells can be achieved through surface modification. This not only protects the therapeutic molecules from enzymatic degradation and improves bioavailability, but also reduces the toxic and side effects caused by systemic administration. Particularly important, inhalable sEV delivery systems enable the loaded immunotherapeutic drugs to specifically accumulate in the lungs, acting directly on lung cancer lesions and the local immune microenvironment, significantly enhancing the specificity and efficacy of immunotherapy, and providing new possibilities for the immunotherapy of refractory cases such as lung cancer brain metastasis.

SEVs are mainly characterized by transmission electron microscopy (TEM), nanoparticle tracking analysis (NTA), fluorescence-activated cell sorting (FACS), and resistive pulse sensing (RPS). For the isolation and purification of sEVs, various methods based on size and density screening have been adopted, including ultracentrifugation, density gradient centrifugation, ultrafiltration, immunoaffinity capture, and precipitation.

Nanovesicles can be engineered to deliver specific cargo and serve as drug delivery agents and targeted therapeutics. Owing to their biocompatibility and lower immunogenicity compared with other drug delivery systems, these bioengineered sEVs are expected to become the future of drug delivery systems to a greater extent. These bioengineered nanovesicles can readily cross lipid bilayers and the blood–brain barrier, and target specific molecules on cancer cells, enabling more precise therapeutic delivery and reducing side effects on healthy tissues. Therefore, targeting cells distinct from those of traditional therapies will be advantageous. Beyond drug delivery, sEVs can also be used in biomarker research, vaccine development, and gene therapy. These vesicles are currently being widely explored for clinical applications, including cancer immunotherapy, neurological disorders, cardiovascular diseases, and others.

Extracellular vesicles can mediate drug resistance by directly exporting or sequestering cytotoxic drugs, thereby reducing their effective concentration at the target site. Extracellular vesicles can also compete with genuine target cells for the binding of immunotherapeutic drugs targeting cellular antigens [168]. EVs can also be used by cancer cells as drug carriers, promoting drug resistance through drug sequestration and excretion [169]. Shedden and colleagues first reported that the expression of genes associated with vesicle shedding was positively correlated with drug resistance in a panel of diverse cancer cell lines [170].

As natural products of the human body, sEVs possess numerous advantages over other synthetic nanocarriers in drug or gene delivery, such as low immunogenicity, high biocompatibility, and high delivery efficiency. In addition, sEVs exhibit excellent stability in circulation, enabling them to travel long distances in vivo and deliver cargo to target cells under both physiological and pathological conditions. Furthermore, sEVs have a cytoplasm-like core, making them suitable carriers for water-soluble drugs [132].

Cancer stem cells are regarded as the seed cells of primary cancers and the root cause of resistance to radiotherapy and chemotherapy. The precise delivery of drugs to cancer stem cells is a key focus and challenge in current cancer therapy. The combination of nanotechnology and small extracellular vesicle (sEV)-based drug delivery is expected to address this challenge and may improve the efficacy and specificity of targeted cancer stem cell therapy. Nie W et al. synthesized small-extracellular-vesicle nanobioconjugates via a biosynthetic approach. Following systemic administration, the conjugates can specifically recognize aCD47 and CD47 on the surface of tumor cells, indicating that nanobioconjugates can actively target tumor cells.

Small extracellular vesicles (sEVs) can also provide solutions to long-standing challenges in the field of drug delivery. Although aspirin has been found to exert antitumor effects, difficulties in delivering it to tumor sites have limited its application. To overcome the problems relating to the poor water solubility of aspirin and the low encapsulation efficiency in small extracellular vesicles, and to further develop novel aspirin-based anticancer drugs, Tran PHL et al. developed and established a nanocrystal small-extracellular-vesicle transport and delivery platform [171].

### 5.3. Exploration and Achievements of Combined Therapy

The autophagy-inhibiting and immunomodulatory small extracellular vesicles (AI-sEVs) studied by Wang et al. serve as a synergistic therapeutic platform targeting tumors, which can overcome the key immune escape mechanisms in non-small cell lung cancer (NSCLC), including autophagy-mediated MHC-I degradation and PD-1/PD-L1 immunosuppression. By delivering IL-7 mRNA to inhibit autophagy and restore MHC-I presentation while concurrently blocking PD-1/PD-L1, AI-sEVs transform immunologically “cold” NSCLC tumors into T cell-inflamed microenvironments. This coordinated strategy significantly outperforms monotherapies, enhancing tumor control and CD8^+^ T-cell activity in preclinical models, highlighting the suppression of autophagy as a critical prerequisite for effective immunotherapy [172].

Although surgery remains the best treatment for patients in the early stage, most patients are diagnosed at an advanced stage and thus are not eligible for resection. In the exploration of treatment technologies, the emergence of targeted therapy has brought about a paradigm shift. After the discovery of epidermal growth factor receptor (EGFR) mutations in 2004, first-generation EGFR tyrosine kinase inhibitors (TKIs), such as gefitinib, have shown significant efficacy in clinical trials [173,174]. Subsequently, various driver gene mutations such as EML4-ALK translocation and KRAS mutation in non-small cell lung cancer (NSCLC) were gradually identified, which have greatly changed the treatment strategies for lung cancer [175]; compared with traditional chemotherapy, it can significantly improve the survival rate of patients with advanced NSCLC who have driver gene mutations [176,177]. However, patients receiving TKI treatment will develop acquired drug resistance, resulting in a limited duration of clinical benefits. Immunotherapy (IO) has become another breakthrough in the treatment of patients with advanced lung cancer, changing the treatment landscape of NSCLC under different circumstances. Multiple large-scale randomized clinical trials have shown that, compared with traditional chemotherapy, immune checkpoint inhibitors (ICIs) can significantly improve the survival rate of NSCLC patients [178,179]. It is a multifaceted process driven by intrinsic tumor mechanisms, microenvironmental crosstalk, and intercellular communication, among which sEVs have become key mediators. These extracellular vesicles not only promote tumor progression and metastasis but also coordinate resistance to chemotherapy, targeted therapy, and immunotherapy by transferring substances such as miRNAs and proteins between cancer and stromal cells [96,180].

Recent studies have shown that sEV targeting methods can complement existing lung cancer therapies by increasing drug sensitivity, overcoming drug resistance, or serving as biomarkers. In the exploration of lung cancer treatment strategies, there are various sEV-combined therapeutic strategies targeting situations such as sEV involvement in drug resistance. The combination of oral sEV-encapsulated paclitaxel and cisplatin can enhance efficacy and reduce toxicity [181]. sEV secretion inhibitors (GW4869) combined with chemotherapy can block sEV-mediated drug resistance and enhance the response to cisplatin. Gefitinib combined with sEV regulation can improve tyrosine kinase inhibitor (TKI) sensitivity by inhibiting sEVs derived from tumor macrophages. Immune checkpoint inhibitors (ICIs) combined with sEV-miRNA-based biomarkers can use sEV miRNAs to monitor ICI response and the development of drug resistance. These strategies provide new directions for addressing problems such as drug resistance in lung cancer treatment.

## 6. Key Challenges Related to Clinical Applications

### 6.1. Difficult Separation and Extraction

Exosomes exhibit heterogeneity in size and molecular content due to their formation and sorting mechanisms. Isolating and enriching exosomes from complex biological components is of great significance for basic research and clinical translation. A variety of isolation methods have been developed to date, and the quantity and purity of exosomes obtained vary significantly. EVs can be isolated from body fluids using multiple techniques, among which ultracentrifugation is the most common and traditional method. However, this centrifuge-based approach has drawbacks, including being time-consuming and prone to degradation, leading researchers to explore alternative isolation methods [182].

To isolate and purify exosomes, a variety of methods based on size and density screening have been adopted, including ultracentrifugation, density gradient centrifugation, ultrafiltration, immunoaffinity capture, and precipitation (Table 2).

### 6.2. The Inhibitor Limitation: Pharmacological vs. Genetic Interrogation

A substantial proportion of functional studies implicating sEVs in resistance relies on GW4869, a neutral sphingomyelinase inhibitor that blocks exosome biogenesis [73,201]. While valuable as a pharmacological tool, GW4869 has several limitations that undermine causal inference. First, it does not inhibit all sEV subtypes: Menck et al. demonstrated that GW4869 decreases exosome secretion but concomitantly increases microvesicle release [73]. Second, GW4869 broadly alters ceramide metabolism and the EV proteome, with extracellular matrix proteins being differentially secreted in a ceramide-dependent manner [73]. Third, and most critically, systemic GW4869 administration inhibits sEV release from all cell types, making it impossible to ascribe observed phenotypes specifically to CAF-derived sEVs. The field urgently requires conditional genetic ablation of sEV biogenesis to interrogate the specific contribution of CAF-derived sEVs in immunocompetent models.

### 6.3. The Concentration Paradox: Dose–Response and Physiological Relevance

Most in vitro sEV supplementation studies use 10–100 μg/mL of purified sEVs, while in vivo tail-vein injections typically administer 10–100 μg of sEVs per mouse. Whether these doses reflect actual sEV concentrations in tumor interstitial fluid remains unknown, as direct measurement within the TME is technically challenging. In NSCLC patients, circulating sEV PD-L1 levels are elevated in EGFR-mutant cases and correlate inversely with CD8^+^ T-cell infiltration, providing clinical evidence that at least some sEV populations reach pathophysiologically meaningful concentrations [71,202]. Mechanistic studies further support this notion: CAF-derived exosomes confer cisplatin resistance through PUM2-dependent packaging, in which dose-dependent rescue by specific molecule knockdown validates the concentration–response relationship [77], while CAF-secreted sEVs remain largely unaltered in release rate even after radiation exposure, suggesting that basal sEV output from CAFs is constitutively sustained in the TME [203]. Nevertheless, if functional effects are observed only at supraphysiological doses, their biological relevance may be overstated. Future studies should incorporate dose–response analyses and, where feasible, estimate local sEV concentrations in tumor tissue to establish in vivo relevance.

### 6.4. The Bidirectionality Problem: Chicken or Egg?

An often-overlooked challenge is directionality. Most studies demonstrate that CAF-sEVs from resistant tumors can transfer resistance to sensitive cells, but this design cannot distinguish whether sEV changes are a cause or a consequence of resistance. Tumor-derived sEVs can reciprocally reprogram normal fibroblasts into pro-tumorigenic CAFs, establishing a bidirectional communication loop that accelerates disease progression [4]. In HNSCC models, POSTN^+^ CAF-derived sEVs were shown to drive macrophage M2 polarization, but whether the POSTN^+^ CAF phenotype itself emerged as a cause or consequence of ICB resistance remains unresolved. Moreover, EGFR-mutant NSCLC cells have been shown to actively secrete sEV PD-L1—a process further enhanced by TKI treatment—suggesting that therapeutic pressure itself can alter sEV cargo profiles and confound causal interpretation [202]. The emergence of resistance may therefore reprogram both tumor cells and CAFs, altering their sEV output in ways that current cross-sectional studies cannot resolve. Longitudinal sampling studies tracking sEV composition before, during, and after resistance development would establish temporality—a key Bradford Hill criterion—but such datasets remain virtually absent from the literature.

### 6.5. Future Directions

The literature reviewed herein establishes that CAF-derived sEVs carry a rich repertoire of immunomodulatory cargoes and can, under defined experimental conditions, suppress anti-tumor immunity [75,76,77]. However, a critical gap separates the current state of mechanistic knowledge from evidence of direct, independent causality in vivo [73]. Addressing this gap will require a concerted effort across three fronts.

First, a transition from correlative pharmacology to cell-type-specific genetics. The deployment of conditional knockout models—in which sEV biogenesis is selectively ablated in CAFs within immunocompetent tumor-bearing mice—represents the most definitive approach to testing causality. Such models would allow researchers to ask whether the loss of CAF-derived sEVs alone, without altering other CAF functions, enhances the efficacy of immune checkpoint blockade or prevents acquired resistance. Proof-of-concept for CAF-targeted genetic intervention exists: fibroblast-specific deletion of Igf2 augments CD8^+^ T-cell infiltration and sensitizes tumors to immunotherapy [204], and NOX4 inhibition normalizes CAFs to restore immunotherapy response in CAF-rich murine tumors [82]. Gar [202] demonstrated sEV secretion and restored CD8^+^ T-cell function in vivo—a phenotype rescued by exogenous sEV administration—providing a template for CAF-specific genetic studies [80].

Second, the application of advanced in vivo tracking technologies. To distinguish paracrine sEV signaling from other modes of intercellular communication, techniques such as Cre-LoxP-based sEV tracking (e.g., CD63-eGFP^fl/fl^ reporters) [205], bioorthogonal metabolic labeling of sEVs [206], or single-vesicle imaging in live tumor tissue will be essential. These approaches can reveal which immune cells take up CAF-sEVs in situ, at what frequency, and with what functional consequences, thereby bridging the gap between in vitro mechanisms and in vivo reality.

Third, longitudinal clinical studies incorporating sEV liquid biopsy. To establish temporality—whether sEV changes precede, coincide with, or follow the development of resistance—prospective cohorts with serial plasma collection before and during immunotherapy are needed. Circulating sEV PD-L1 has been longitudinally tracked and shown to correlate with tumor burden and treatment response in NSCLC patients [202,207], and sEV miRNA signatures have demonstrated prognostic value for chemo-immunotherapy outcomes [70]. Standardized sEV isolation and characterization protocols, compliant with MISEV2023 guidelines [40], should be adopted to enable cross-study comparisons and meta-analyses.

By explicitly acknowledging the current evidentiary limitations and charting a path toward rigorous causal demonstration, this review aims not only to summarize what is known but also to catalyze the next generation of experiments that will definitively establish—or refine—the role of CAF-derived sEVs in lung cancer immunotherapy resistance.

## 7. Conclusions

CAF-derived sEVs carry diverse immunomodulatory cargoes that can suppress T-cell and NK cell function, drive M2 macrophage polarization, and activate pro-resistance signaling pathways. These mechanistic findings, together with clinical associations between sEV cargoes and poor immunotherapy outcomes, strongly implicate CAF-sEVs in resistance biology. However, direct in vivo causal evidence—distinguishing sEV-specific effects from those of other CAF-derived factors—remains lacking. Key obstacles include reliance on broad-spectrum inhibitors and the absence of CAF-specific genetic models. Future priorities include: (i) employing conditional knockout models to selectively ablate sEV biogenesis in CAFs; (ii) applying in vivo tracking technologies to resolve sEV uptake by specific immune populations; and (iii) conducting prospective clinical cohorts with standardized sEV liquid biopsy to validate biomarkers and establish temporality with resistance onset. Addressing these gaps will determine whether CAF-sEVs are causal mediators, contributing modifiers, or biomarkers of immunotherapy resistance—a distinction critical for therapeutic targeting.

## Figures and Tables

**Table 1:** Biomarkers of small extracellular vesicles. Summarizes lung cancer-specific studies reporting associations between CAF-sEV cargoes and clinical outcomes, providing correlative evidence that supports a causal role in immunotherapy resistance.

Types	SEV-Associated Factors	Functions	Expression Level	Reference
Proteins	EGFR	Induces tolerant dendritic cells and helps tumor escape immune surveillance, which is related to drug resistance of lung cancer	Upregulated	Xu et al. [150]
	CD151	PI3K/Akt-EMT axis drives cisplatin and targeted-drug resistance in lung cancer	Upregulated	Sandfeld-Paulsen et al. [151]
	tetraspanin 8	TME remodeling, EMT induction and anti-apoptosis initiation are the basis of anti-chemotherapy and targeted therapy for lung cancer	Upregulated	Liu et al. [152]
	MRP2	Efflux-mediated drug extrusion enhances chemoresistance and renders lung cancer cells insensitive to chemotherapy	Upregulated	Filipits et al. [153]
	PD-L1	Suppresses T-cell activity, enabling tumor immune evasion and immunotherapy resistance.	Upregulated	Yu et al. [154]
	STAT3	Induces EMT, promoting lung cancer metastasis and drug resistance	Upregulated	Mohrherr et al. [155]
	LDHA	Rewires TME metabolism to blunt immune response and confer resistance in lung cancer	Upregulated	Wang et al. [156]
	Galectin-9	Suppresses CD8^+^ T-cell activity via TIM-3 engagement, driving immune evasion and immunotherapy resistance in lung cancer	Upregulated	Chen et al. [157]
	IL-6	In non-small cell lung cancer, IL-6 promotes TGF-β-induced EMT and cisplatin resistance	Upregulated	Shintani et al. [158]
RNAs	miR-21-5p	Targets tumor-suppressor PTEN, activates PI3K/Akt signaling, and enhances cisplatin-gemcitabine chemoresistance in lung cancer	Upregulated	Zhong et al. [159]
	miR-20a	Promotes the proliferation of NSCLC by upregulating programmed death-ligand 1 (PD-L1) and inhibiting PTEN	Upregulated	Shi et al. [76]
	miR-155-5p	Suppresses SOCS1, activates JAK/STAT3 signaling, and drives erlotinib resistance in NSCLC	Upregulated	Pang et al. [160]
	miR-451a	Reduced expression relieves ABCB1 (P-glycoprotein) suppression, boosts drug efflux, and confers cisplatin resistance in lung cancer	Downregulated	Reis et al. [161]
	circKIF20B circRNA CDR1as	Suppresses gefitinib resistance and cell proliferation in non-small cell lung cancer Weakened “molecular-sponge” sequestration of miR-21, hyper-activates the PI3K/Akt axis, and drives gemcitabine resistance in lung cancer	Upregulated Upregulated	Wei et al. [162] Zhao et al. [163]
	Circ_0001421	Promoted cell proliferation, migration, invasion and glycolysis in LC by regulating the miR-4677-3p/CDCA3 axis, which provides a new mechanism for LC tumor progression	Upregulated	Zhang et al. [164]
	lncRNA OIP5-AS	Export OIP-AS to suppress miR-142-5p and induce PD-L1 expression, EVs can be involved in immune tolerance of tumors	Upregulated	Jiang et al. [74]
	lncRNA LINC01614	Enhance glutamine uptake in lung adenocarcinoma	Upregulated	Liu et al. [165]
	lncRNA H19	Sponges miR-let-7 family, relieves HMGA2 suppression, promotes EMT, and mediates cisplatin and EGFR-TKI resistance in lung cancer	Upregulated	Chen et al. [166]
	PITPNA-AS1	Acts as an oncogene to promote lung cancer cell proliferation and migration.	Upregulated	Chen et al. [167]

**Table 2:** Isolation techniques of small extracellular vesicles.

Techniques	Methods	Advantages	Disadvantages	Prominent Examples	Refs.
**Conventional techniques**					
Isolation techniques of small extracellular vesicles	Differential ultracentrifugation	Cashback as early as possible	engthy process; large sample volume; requires ultracentrifuge	It was first proposed by Johnstone et al. and used to isolate exosomes from reticulocyte culture media.	[180]
	Gradient density ultracentrifugation	A better alternative beyond achieving higher purity	Time-consuming processes and the demand for large quantities of biological fluids	Sucrose gradient-purified prostasomes.	[43]
Filter	Ultrafiltration	Simple operation and efficient purification	Low yield	Nita N Böing and others developed an SEC-based protocol.	[183,184]
	Size Exclusion Chromatography (SEC)	Mild treatment and non-destructive results; the combination of SEC and ultracentrifugation may improve recovery and purity	Lacks specificity; too much contamination	Using cross-linked Sepharose CL-2B columns, exosomes with a diameter greater than 70 nanometers can be efficiently isolated from platelet-free supernatant.	[185,186]
Precipitation technology	Precipitation technology	A relatively high exosome yield	High cost and easy contamination of coprecipitated protein aggregates	Rider et al. proposed an exosome purification method called ExtraPEG, which enables rapid enrichment of exosomes and harvests sufficient contents from vesicles for downstream analysis.	[187,188,189]
**Novel techniques**					
Immunoaffinity/Immunomagnetic Enrichment	Immunoaffinity separation	It enables the dissociation of captured exosomes with high recovery rate and purity.	High cost and reliance on markers	Zhang et al. fabricated anti-CD81 functionalized microfluidic chips for the isolation of exosomes from plasma samples; Kang et al. developed an exosome-specific dual-mode immunofiltration (ExoDIF) device; and Kang et al. proposed an on-demand EV chip (EVOD).	[190,191,192,193]
Magnetic Separation and Concentration	Magnetic separation	Convenience and high efficiency facilitate rapid separation and enable the circulation of exosomes within the required regions.	High-cost; marker dependent	Fang et al. isolated exosomes via CD63 antibody-conjugated magnetic nanoparticles, and novel immunoaffinity superparamagnetic nanoparticles (IS-NPs) have been demonstrated to exhibit high efficiency.	[191,194]
Based on physical characteristics	Microfluidic chip	High yield, capable of examining urine	Contamination of same-sized vesicles; lacks specificity	Liu et al. designed a size-based total exosome isolation chip (ExoTIC), in which exosomes of 30–200 nanometers are enriched and purified through multilayer nanoporous membranes. Sunkara et al. developed a microfluidic tangential flow filtration device, Exodisc, for the isolation of exosomes from human plasma and urine.	[195,196]
Lipid separation	Lipid separation	High capture rate and high efficiency	Contamination of other phospholipid membrane vesicles; lacks specificity	Wan et al. reported a lipid nanoprobe for the rapid separation of exosomes from plasma.	[193,197]
Acoustic-based isolation methods	Microfluidics		Design and fabrication for finer gradations; finer-grade separation of subpopulations	Anson et al. have developed integrated acoustic devices that enable rapid, non-contact and continuous separation of exosomes from urine and plasma samples. Wu et al. have developed an acoustofluidic platform.	[198]
Thermophoretic enrichment	Thermophoretic enrichment	Low-cost and sensitive	Contamination of same-sized vesicles; lacks specificity	Liu et al. developed a sensitive thermophoretic method for the enrichment of tumor-derived exosomes.	[199,200]

## Data Availability

The original contributions presented in this study are included in the article. Further inquiries can be directed to the corresponding author.

## Abbreviations

AC	adenocarcinoma of the lung
APC	antigen-presenting cell
CAF	cancer-associated fibroblast
CAR	chimeric antigen receptor
circRNA	circular RNA
ECM	extracellular matrix
EMT	epithelial–-mesenchymal transition
EV	extracellular vesicle
ICI	Immune checkpoint inhibitor
IFN-α	interferon-alpha
lncRNA	Long non-coding RNA
MVB	multivesicular body
NF	nuclear factor
NSCLC	non-small cell lung cancer
PDGF	platelet-derived growth factor
SCF	stem cell factor
SCLC	small cell lung cancer
sEV	small extracellular vesicle
TGF-β	transforming growth factor-β
TME	the tumor microenvironment
VEGF	vascular endothelial growth factor
